# Cubic Membranes Formation in Synchronized Human Hepatocellular Carcinoma Cells Reveals a Possible Role as a Structural Antioxidant Defense System in Cell Cycle Progression

**DOI:** 10.3389/fcell.2020.617406

**Published:** 2020-12-14

**Authors:** Deqin Kong, Rui Liu, Jiangzheng Liu, Qingbiao Zhou, Jiaxin Zhang, Wenli Li, Hua Bai, Chunxu Hai

**Affiliations:** ^1^Shaanxi Provincial Key Lab of Free Radical Biology and Medicine, The Ministry of Education Key Lab of Hazard Assessment and Control in Special Operational Environment, Department of Toxicology, School of Public Health, Air Force Medical University (Fourth Military Medical University), Xi'an, China; ^2^Frontiers Science Center for Flexible Electronics, Xi'an Institute of Flexible Electronics (IFE) and Xi'an Institute of Biomedical Materials & Engineering, Northwestern Polytechnical University, Xi'an, China

**Keywords:** cubic membranes (CMs), cell cycle, reactive oxygen species (ROS), mitochondria, cell synchronization

## Abstract

Cubic membranes (CMs) represent unique biological membrane structures with highly curved three-dimensional periodic minimal surfaces, which have been observed in a wide range of cell types and organelles under various stress conditions (e. g., starvation, virus-infection, and oxidation). However, there are few reports on the biological roles of CMs, especially their roles in cell cycle. Hence, we established a stable cell population of human hepatocellular carcinoma cells (HepG2) of 100% S phase by thymidine treatment, and determined certain parameters in G2 phase released from S phase. Then we found a close relationship between CMs formation and cell cycle, and an increase in reactive oxygen species (ROS) and mitochondrial function. After the synchronization of HepG2 cells were induced, CMs were observed through transmission electron microscope in G2 phase but not in G1, S and M phase. Moreover, the increased ATP production, mitochondrial and intracellular ROS levels were also present in G2 phase, which demonstrated a positive correlation with CMs formation by Pearson correlation analysis. This study suggests that CMs may act as an antioxidant structure in response to mitochondria-derived ROS during G2 phase and thus participate in cell cycle progression.

## Introduction

It is universally known that all organisms except viruses have biomembranes. The emergence of biomembranes, which enable cells to exist independently of environment, is a leap in biological evolution. Generally, the morphology of biomembranes is a bimolecular lamellar structure. However, there are also non-lamellar membrane structures in the cells, such as the cubic membranes (CMs), which have highly curved 3D nanoscale periodic structure with a 3-fold periodic minimum surface (Almsherqi et al., 2012; Paillusson et al., 2016; Deng et al., 2017). CMs seem to be evolutionally conserved and have been continually discovered by transmission electron microscopy in all the five kingdoms of life, namely monera, protista, fungi, plant, and animalia (Almsherqi et al., 2009; Zhan et al., 2017).

Although the precise biological role of CMs is unclear until now, research in recent decades has provided some clues. Chong et al. found that in the case of starvation, the inner membrane of mitochondria of amoeba was transformed into a cubic membrane structure to enhance mitochondrial function and maintain ATP production-a process critical for survival (Chong et al., 2018). The formation of cubic mitochondrial cristae in amoeba cells protects them against oxidative damage by enhancing the leakage of H_2_O_2_ and reactive oxygen species (ROS) in mitochondria and reducing the sensitivity of membrane lipids to oxidants (Deng et al., 2002). In addition, it is found that phospholipids rich in long chain polyunsaturated fatty acids in CMs can preferentially bind with superoxide anions, thus inhibiting the oxidative damage of RNA induced by ROS (Deng and Almsherqi, 2015). Another role of CMs is a “virus factory” in the host cells infected by virus. Virus infection can lead to rearrangement of cell biomembrane system and induce appearance of CMs, which is likely to provide a protective microenvironment for virus assembly and proliferation (Deng et al., 2010).

Of particular concern is the potential role of CMs in cell cycle progression. In 1965, an observational study found that a large number of CMs from mitochondria appeared during mitosis in amoebae (Daniels et al., 1965). Although the role of CMs is unknown, the study suggests the possibility that CMs may get involved in cell division. Since then, however, this phenomenon seems not to be confirmed in mammalian cells, particularly in the cells derived from humans. Hence, by employing the universally accepted cell synchronization method (Banfalvi, 2017), we tried to investigate the changes of CMs in different cell cycle phases of synchronized HepG2 cells. Our study demonstrated the appearance of CMs at G2 phase of cell cycle in mammalian cells, and by evaluating the levels of mitochondrial ROS and ATP, we conducted further research on the potential roles of CMs in cell cycle.

## Materials and Methods

### Cell Culture

HepG2, human hepatocellular carcinoma cell line, was obtained from the American Type Culture Collection (ATCC) and cultured with RPMI 1640 medium (HyClone) containing 10% fetal bovine serum at 37°C in 5% CO_2_ and saturated humidity. When the confluence reached 80%, the cells were digested with 0.25% trypsin (HyClone) for generation or subsequent experiments.

### The Acquisition of Synchronized Cells

HepG2 cells were seeded in 6-well plates, and when the adherent rate was about 50%, the cells were treated with 1 mM thymidine (Sigma, T1895) for 48 h. The cells were washed with warm fresh medium twice and 25 μM deoxycytidine (Sigma-Aldrich) in medium was added to promote release. The cells in G2/M and G1 phases were collected at indicated time points.

### The Detection of Cell Cycle Distribution

The cell cycle detection kit (KeyGEN BioTECH, KGA512) was used to detect cell cycle distribution according to the instructions. Briefly, the cells were fixed with 70% ethanol and stored at 4°C overnight. Then, the fixed cells were centrifuged at 1,000 g for 5 min. After being washed with the PBS buffer, the cell samples were incubated with 100 μL RNAase at 37°C for 30 min and then with 400 μL PI at 4°C for 30 min under a dark condition. The flow cytometry (BD Biosciences) was used to detect fluorescence signal at FL2-A channel by the CFlow Plus software. A quantitative analysis of the proportion of each phase in a cell cycle was conducted by the LT ModFit software.

### The Detection of Cell Apoptosis

Cell apoptosis was detected by the AnnexinV-FITC/propidium iodide (PI) staining (Mao et al., 2019). When the treatment was completed, the cells were harvested and washed twice with PBS. After centrifugation, cells were resuspended at 2 × 10^5^/mL and assayed by the AnnexinV-FITC/PI kit in accordance with the manufacturer's instructions. In each sample, 500 μL binding buffer, 5 μL Annexin V-FITC and 5 μL PI were added in turn and incubated at a room temperature for 15 min under a dark condition. Apoptosis was detected by flow cytometry (BD Biosciences). Annexin V-FITC and PI fluorescents were detected by FL1 and FL3 channels, respectively.

### The Ultrastructural Study by Transmission Electron Microscope

HepG2 cells were seeded in T75 cell culture flask. After synchronization and release at indicated time points, the cells were collected and fixed by 2.5% glutaraldehyde. After ultrathin sections were made, the ultrastructure was observed and the number of CMs in cells was counted by transmission electron microscope (Tecnai G2, FEI).

### The Determination of ROS

The intracellular ROS level was determined by DCFH-DA staining as described (Kong et al., 2019). After the cells were collected and washed with PBS, DCFH-DA dye (Sigma, D6883) with a final concentration of 10 μmol/L was used to incubate with each sample for 30 min at 37°C under a dark condition. Intracellular ROS levels were detected by flow cytometry (BD Biosciences) (EX/EM: 488 nm /530 nm). The CFlowPlus software was used to collect and process the data.

### The Measurement of Mitochondrial ROS (mtROS)

A mitochondria-targeted MitoSOX probe was used to measure mtROS levels as described (Gao and Zhang, 2020). After the cells were collected and washed with PBS, each sample was added into 5 μmol/L MitoSOX (Invitrogen, M36008). After incubation for 30 min at 37°C under a dark condition, the flow cytometry (BD Biosciences) was employed to detect fluorescence intensity at FL2A channel (EX/EM: 510 nm /580 nm).

### The Detection of ATP Content

Enhanced ATP Assay Kit (Beyotime Biotechnology, S0027) was employed to detect the ATP content as described (Ding et al., 2020). After lysis of the cells on the ice, the lysate was centrifuged at 4°C for 5 min and the supernatant was taken to store at 4°C. One hundred microliter ATP-detection solution was added to the test wells for 5 min. Twenty microliter samples or standard products were added to the test wells and RLU values were detected by multifunctional microplate reader (Tecan Group Ltd.). The concentrations of ATP in the samples were calculated by the standard curve. The protein concentration was determined by BCA assay kit (Thermo Scientific) and ATP content was normalized by protein concentration.

### Statistical Analysis

The values were expressed as the mean ± SD. Statistical comparison was estimated by a one-way ANOVA followed by a Dunnett-*t* test for comparing all the groups with the control group. All analyses were performed using the Statistical Package for the Social Sciences 20.0 (SPSS20.0) software. In all the cases, a two-tailed p-value lower than 0.05 was considered statistically significant.

## Results

### The Highly Synchronized Cells Were Induced by Thymidine Treatment

**Figure 1:**
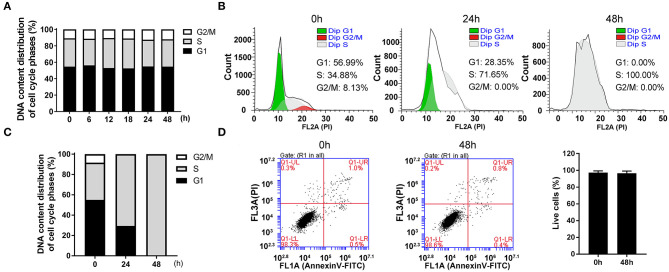
The highly synchronized cells in S phase were induced by thymidine for 48 h. **(A)** The distributions of cell cycle at indicated time after seeding asynchronous HepG2 cells. **(B)** Flow cytometric analysis showed the changes of cell cycle distributions after treatment with 1 mM thymidine for 0, 24, and 48 h, and the percentages of cells in G1, S and G2/M phases were statistically shown in **(C)**. **(D)** After treatment with 1 mM thymidine for 48 h, AnnexinV-FITC/PI staining was employed to assess cell apoptosis, and the percentage of live cells was shown in the histogram. All data were denoted as the means ± SD; *N* = 3.

The cultured cells *in vitro* were a mixture of cells in all phases. By collecting the cells at indicated times after seeding, we found that the percentage of all phases remained relatively constant and the cells in G1, S and G2/M phases accounted for about 54.87, 36.22, and 8.90%, respectively (Figure 1A).

In order to collect synchronized cells, we screened a series of synchronization methods and employed thymidine treatment as a reversible blocking agent to induce cell synchronization. After treatment with 1 mM thymidine for 24 and 48 h, the distribution of cell phases showed a significant change in a time-dependent manner (Figures 1B,C). At 24 h, the cell proportion in G1 phase decreased and the cell proportion in G2/M phase reached nearly zero. The cells in S phase markedly increased to about 70%. At 48 h, the unique peak (i.e., S peak) appeared and the percentage of cells in S phase reached 100%. As an ideal synchronization method should not cause obvious apoptosis, we thus examined cell apoptosis after treatment with thymidine for 48 h. As shown in Figure 1D, the percentage of live cells at 48 h was about 98%, which was roughly the same as that at 0 h. Thus, treatment with thymidine for 48 h did not induce apoptosis, suggesting that thymidine treatment was a reasonable and reliable method for cell synchronization.

### The Cells in G2, M and G1 Phases Were Collected After S Phase-Blocking Cells Were Released

**Figure 2:**
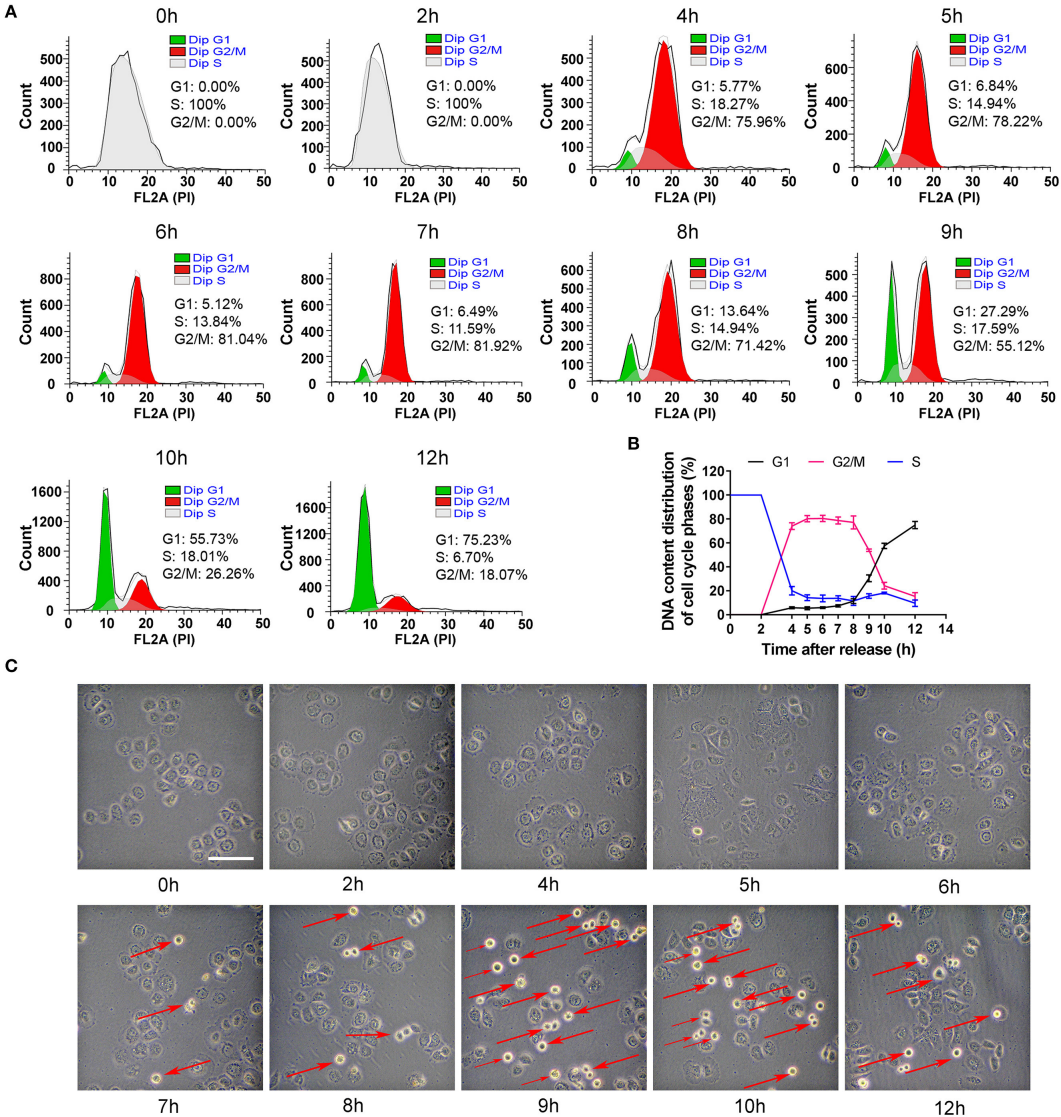
The distribution changes of cells in G2, M and G1 phase after S phase-blocking cells were released. **(A)** The distributions of cell cycle phases were measured by flow cytometry at indicated time points after releasing from S phase, and the dynamic changes of G1, S and G2/M phases were shown in **(B)**. All of the values were denoted as the means ± SD; *N* = 3. **(C)** Representative photos showed the changes of general morphology of cells at indicated time points. The red arrow marking round cells represents those undergoing mitosis. The scale is 100 μm.

To collect the cells in other phases, the S phase-blocking cells were released and then DNA contents were determined at different time points (0, 2, 4, 5, 6, 7, 8, 9, 10, and 12 h). As shown in Figures 2A,B, the distribution of cell phases at 2 h was the same as that at 0 h. However, the phase peaks dramatically altered 2 h later. The cells in G2/M phase robustly increased to 75% at 4 h, reached nearly the maximal level (~80%) at 6–7 h. At 8 h, the percentage of G2/M phase slightly decreased. At 9 h, the cells in G2/M phase decreased dramatically and G1 peak suddenly rose, and the cells in G1 phase were about 30% in cell population. More than half of the cells were in G1 phase at 10 h and the synchronization rate of 75% was achieved at 12 h. Based on an analysis of the time-dependent changes of cell cycle, G2 phase of cell cycle was from 4 to 8 h, and M phase was taken about 2 h from 8 to 10 h, which was verified by morphology showing that the round cells (undergoing mitosis) substantially appeared at 9 h (Figure 2C). Additionally, cells entered G1 phase at 12 h.

### CMs Formed in the Cells of G2 Phase

**Figure 3:**
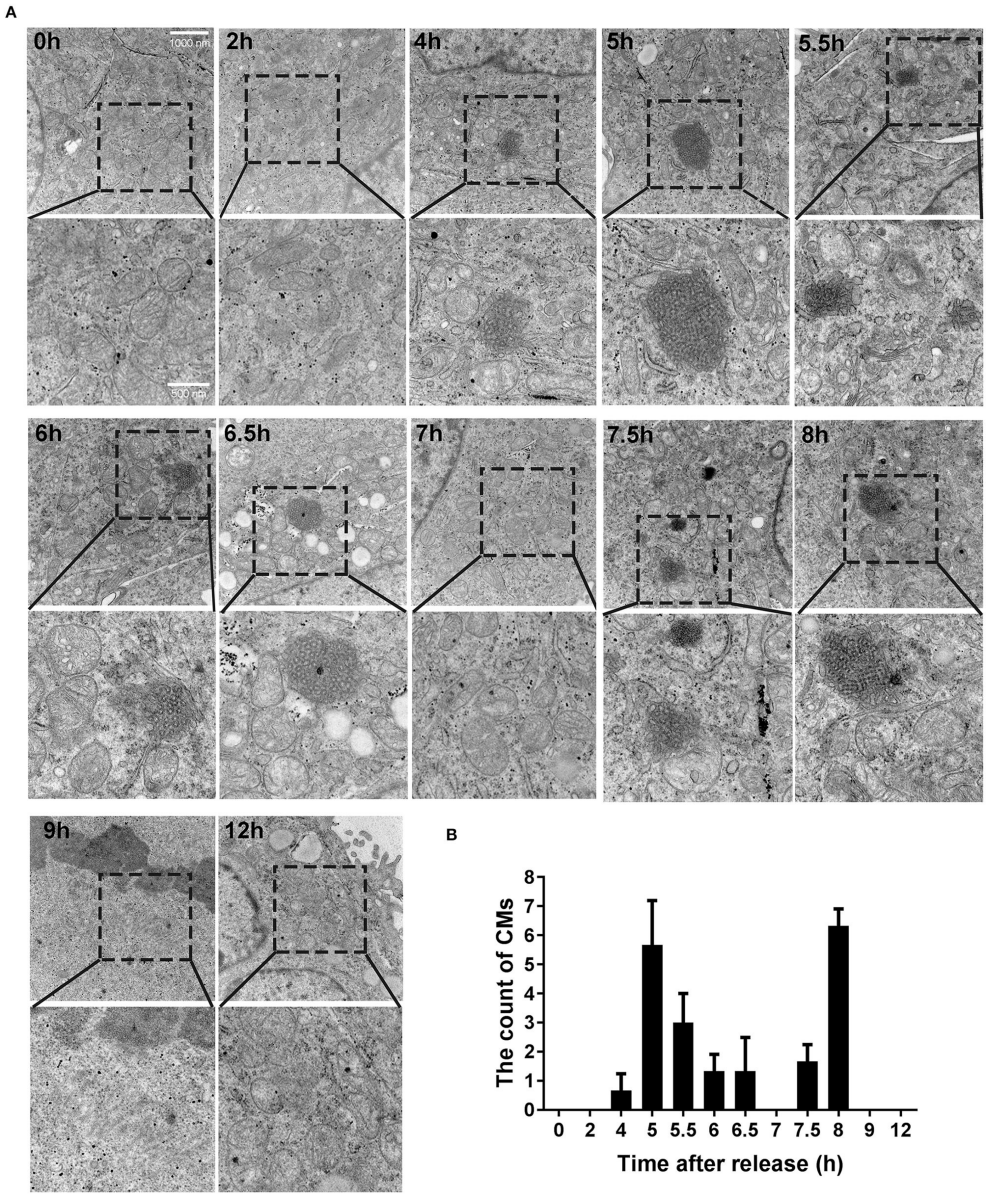
CMs appeared in G2 phase of cell cycle. **(A)** Ultrastructural observations of cells at indicated time points after releasing from S phase. Representative images (20500X magnification) by transmission electron microscope were shown and the enlarged images in the rectangular area were placed below, respectively. **(B)** Statistics of CMs in every 100 cells at indicated time points. All values were denoted as the means ± SD; *N* = 3.

Based on the results presented above, we identified some more targeted time points (0, 2, 4, 5, 5.5, 6, 6.5, 7, 7.5, 8, 9, and 12 h), trying to observe CMs by transmission electron microscope. As shown in Figure 3A, CMs weren't observed at 0 h and 2 h. However, different sizes of CMs were observed from 4 to 8 h except 7 h (i.e., in G2 phase). At 9 and 12 h, CMs were in absence. In conclusion, CMs formed in G2 phase of cell cycle, and they were more likely to be observed in cells at 5 and 8 h, which showed a bimodal distribution (Figure 3B).

### The Formation of CMs Was Positively Correlated With ROS Level

**Figure 4:**
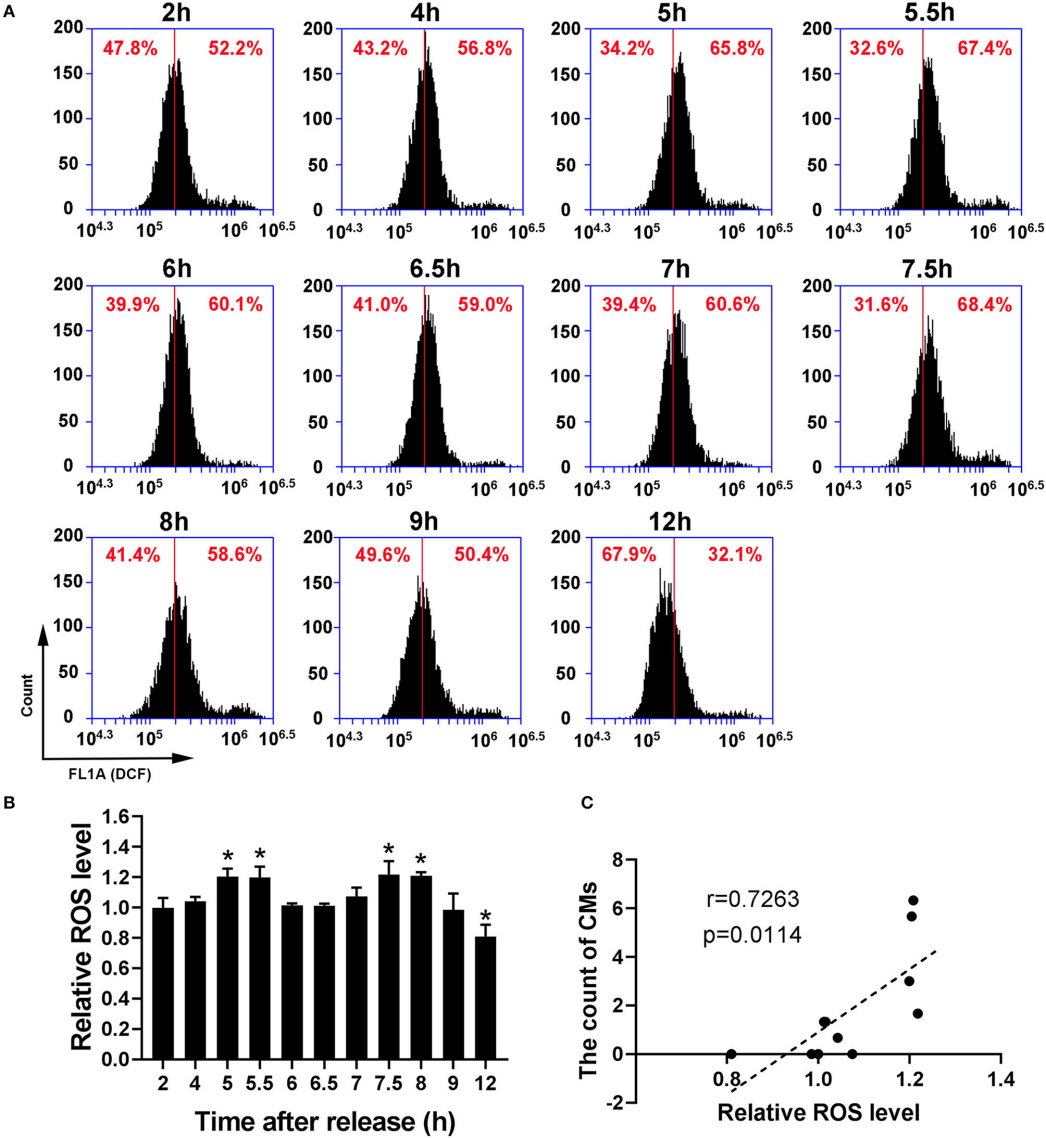
The occurrence of CMs in G2 phase was correlated with ROS level. **(A)** Representative charts showing the change of ROS peaks after releasing from S phase which were detected by flow cytometry. The middle line was as a reference. **(B)** The histogram represented the change of ROS levels at indicated time points by analysis of fluorescence intensity. All values were denoted as the means ± SD; *N* = 3. *P ≤ 0.05, compared to the 2 h group. **(C)** The correlation between the average count of CMs and relative ROS level was shown by Pearson's correlation analysis.

**Figure 5:**
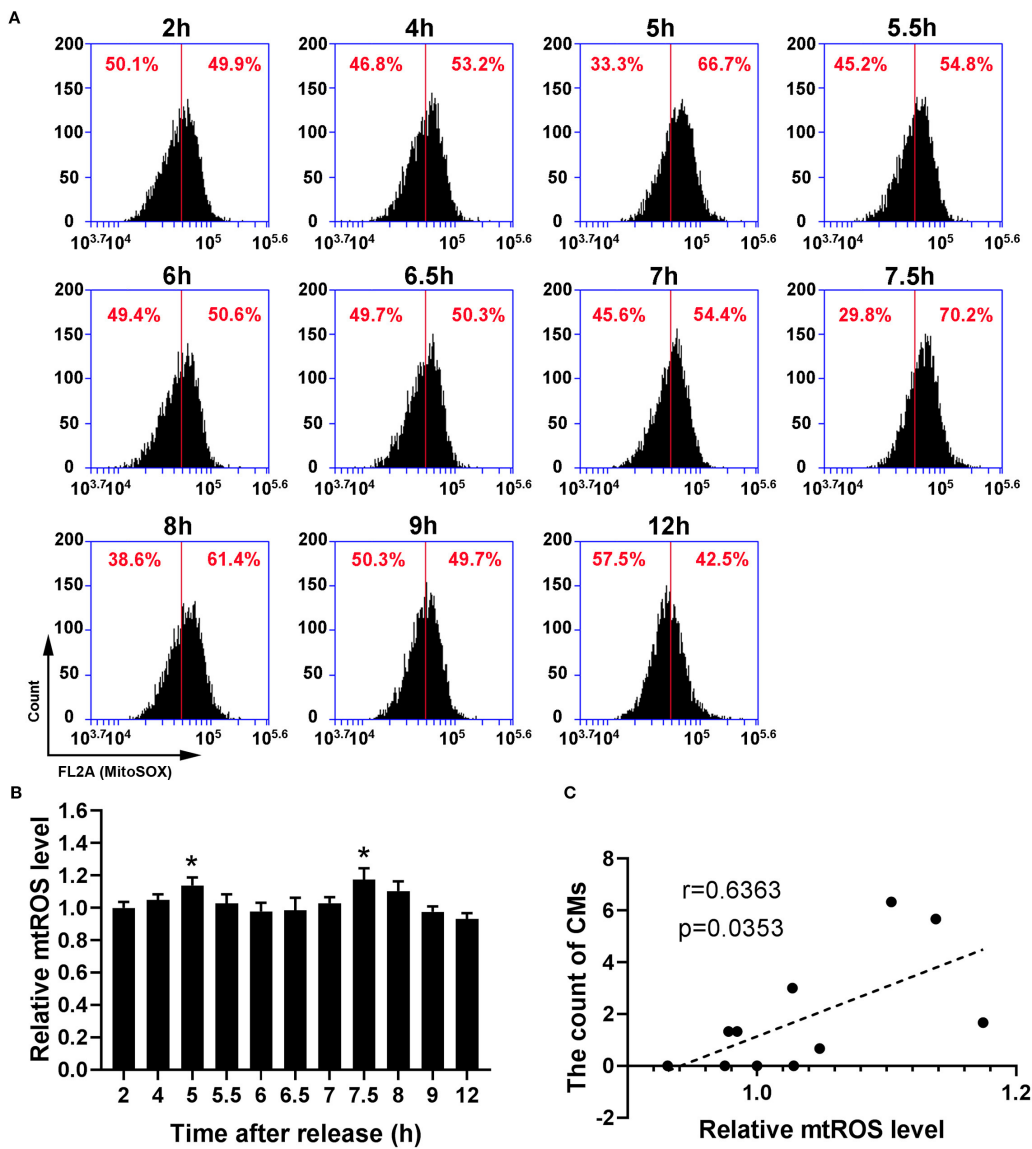
The presence of CMs in G2 phase was correlated with mtROS level. **(A)** Representative charts showing the shift of mtROS peaks after releasing from S phase which were detected by flow cytometry, and the middle line was as a reference. **(B)** The change of mtROS levels at indicated time points was shown by analysis of fluorescence intensity. All data were expressed as the means ± SD; *N* = 3. *P ≤ 0.05, compared to the 2 h group. **(C)** The correlation between the average count of CMs and relative mtROS level was shown by Pearson's correlation analysis.

Because recent studies show that CMs correlate with cellular antioxidant activity, we measured the levels of intracellular ROS and mtROS. As shown in Figures 4A,B, intracellular ROS level showed a dynamic change with bimodal distribution and significantly increased at 5–5.5 h and 7.5–8 h. Then, we conducted a Pearson' correlation analysis to identify the relationship between CMs formation and intracellular ROS level, and found a positive correlation (*r* = 0.7263, *p* < 0.05, Figure 4C). Similarly, mtROS level rose at 5 and 7.5 h (Figures 5A,B), demonstrating a positive correlation with CMs formation (Figure 5C).

### The Presence of CMs Had a Positive Correlation With ATP Level

**Figure 6:**
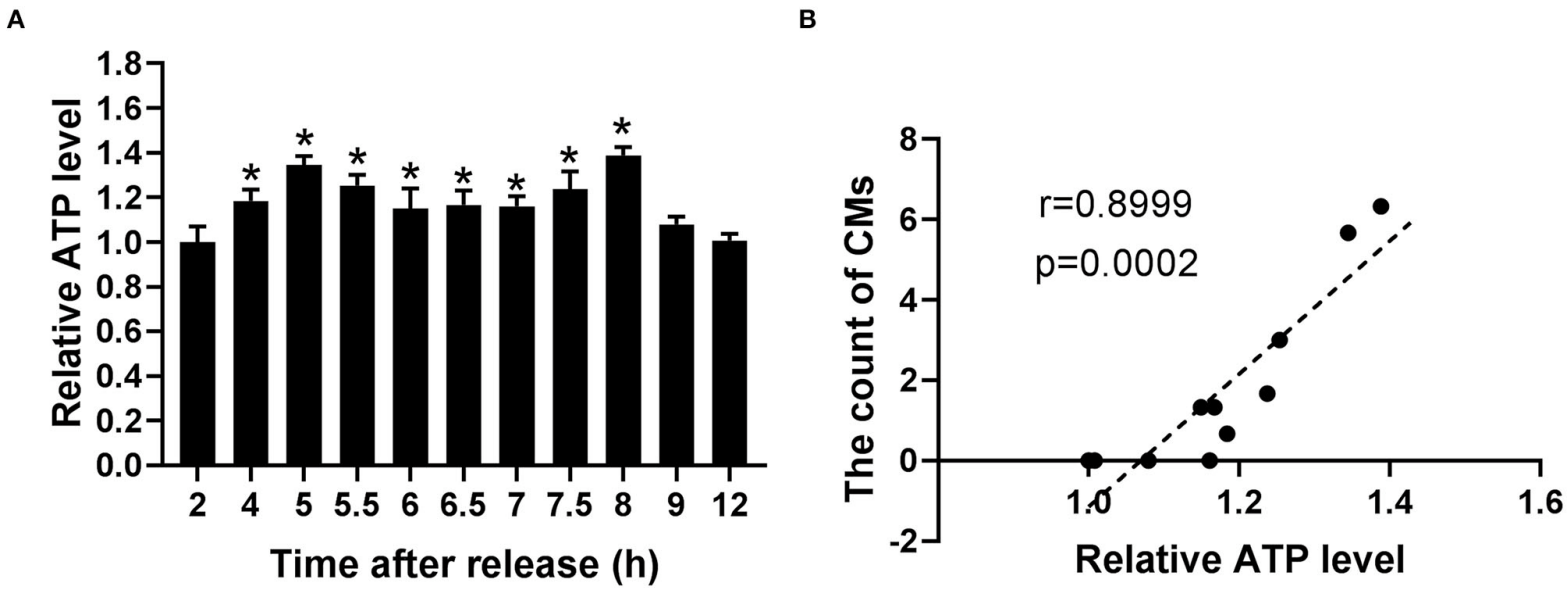
The occurrence of CMs in G2 phase was correlated with ATP level. **(A)** Histograms represented the change of ATP level at indicated time points after release. All of the values were denoted as the means ± SD; *N* = 3. *P ≤ 0.05, compared to the 2 h group. **(B)** Pearson's correlation analysis showed the correlation between the average count of CMs and relative ATP level.

Because of the relationship of CMs with mtROS and their potential association with mitochondrial function, ATP levels were detected to assess the mitochondrial function at indicated time. According to Figure 6A, the ATP levels significantly increased within the range from 4 to 8 h, reaching the peak levels at 5 and 8 h. Moreover, Pearson's correlation analysis showed a positive correlation between CMs formation and ATP level (*r* = 0.8999, *p* < 0.001, Figure 6B).

## Discussion

Although CMs are found in many cell types, there still lacks a comprehensive understanding of their biological role. The role of CMs in cell cycle has been of interest since the discovery of CMs in amoeba cells during mitosis in 1965. However, there seems to be no evidence for the existence of CMs in mammalian cell cycle. This study proves that CMs exist in the mammalian (more specifically in human) cell cycle and only in G2 phase, and that CMs are associated with mitochondria-derived ROS. It is thus suggested that CMs, as a structural antioxidant defense system, may play an important role in cell cycle.

Generally, proliferating cells go through four phases, G1, S, G2, and M, among which G2/M phase takes the least time (Mercadante et al., 2020). Therefore, the proportion of G2/M cells is the lowest, for example, only 8.9% in HepG2 cells (Figure 1A). Obviously, the observation of events in G2/M phase requires cell synchronization, a technique of driving cells into one phase by certain drugs or other methods (Banfalvi, 2017). In this study, we used thymidine treatment as a synchronization method, which is widely used in cell cycle studies (Banfalvi, 2017; Booy et al., 2017). The ideal synchronization cannot cause damage to cells, and the cells can continue to complete mitosis after the synchronization treatment was removed. The synchronization method employed in this study was suitable, as it did not cause apoptosis or abnormal cell division (Figures 1D, 2C). It was due to the application of cell synchronization that in this study CMs were observed during G2 phase of the mammalian cell cycle. Compared with the CMs discovered in amoeba cells in G2 phase by Daniels et al. (1965), the CMs discovered in this study seem to have a similar structure (parallel, wavy or zig-zag configuration) and also appear in G2 phase, suggesting that CMs may be conservative in cell cycle. Compared with the method to roughly determine cell phase by cell morphological observation applied by Daniels et al. (1965), the synchronization method in this study can provide more details about CMs, such as the higher possibility of CMs to appear in the early and late G2 phase. Therefore, cell synchronization method was needed in the study of CMs in cell cycle.

It is well-known that ROS have both beneficial and deleterious effects, and “eustress” is employed to describe physiological stress (Sies, 2017; Antonioni et al., 2020; Sies and Jones, 2020). Obviously, the increase in ROS level in G2 phase of cell cycle belongs to eustress. However, the mechanism by which the high level of ROS does not cause oxidative damage remains unclear. This study found that the occurrence of CMs in G2 phase was closely related to the increased ROS, suggesting that CMs may be involved in cell cycle as an antioxidant. In fact, oxidant treatment has been reported to induce CMs formation (Sankhagowit et al., 2016). Although there is no report on the antioxidant effects of CMs in mammals, Deng et al. found that starvation-induced CMs in the amoeba *Chaos* protected RNAs and the gene expression regulatory system from oxidative damage caused by fasting, suggesting that CMs may play a protective role as an antioxidant and further promote cell survival under starvation-induced stress (Deng and Almsherqi, 2015; Deng et al., 2017; Chong et al., 2018). CMs are considered as an antioxidant defense system in organisms because of an abundance of plasmalogens with antioxidant properties in CMs (Deng and Almsherqi, 2015; Luoma et al., 2015). Plasmalogens are kinds of glycerophospholipids containing ether bonds, which are widely distributed in mammalian tissues, especially in heart, brain and muscle tissues (Paul et al., 2019). It is reported that plasmalogens can play a protective role against oxidative damage due to the special molecular composition and spatial conformation, and the supplementation of plasmalogens has a potential treatment effect on cardiometabolic and neurodegenerative diseases, such as Alzheimer's disease and Parkinson's disease (Luoma et al., 2015; Zhou et al., 2020). Hence, there is a high possibility that the appearance of CMs in G2 phase of cell cycle provides a shelter for RNAs which is critical to G2/M transition and mitosis, in case of attack by elevated ROS.

Mitochondrion is an energy factory of cells. When it produces ATP, some electrons in the electron transport chain of mitochondria inevitably leak out to produce mtROS (Figueira et al., 2013). Although the source of intracellular ROS includes but is not limited to mitochondria, peroxidase and NADPH oxidase complex under certain pathological condition, mitochondria are considered to be the most important ROS source in mammalian cells (Mailloux, 2020; Mari et al., 2020). MnSOD located in mitochondria is an important antioxidant enzyme. The methylation-dependent conformational change of MnSOD can gradually reduce its activity from G1 phase to G2/M phase, resulting in the increase in superoxide anion content in G2/M phase, which may be an important reason for the rise of ROS in G2 phase (Sarsour et al., 2012). This study found that mtROS (by employing mitochondria-targeted probe MitoSOX) and ATP levels increased in G2 phase, showing a similar trend as ROS. Moreover, the increase in mtROS level is correlated with the emergence of CMs, suggesting that the enhanced mitochondrial function (providing energy for mitosis) in G2 phase may lead to the increase in ROS level and the subsequent CMs formation. Accumulating evidence suggests that mitochondrial function has a dynamic change in cell cycle (Owusu-Ansah et al., 2008; Horbay and Bilyy, 2016; Mascanzoni et al., 2019), and ATP production is much more dependent on mitochondrial respiration in G2/M phase of cell cycle (Bao et al., 2013). This may be due to cell cycle machinery cyclin B1/Cdk1, which localized to the matrix of mitochondria and phosphorylated a cluster of mitochondrial proteins, and eventually resulted in the enhanced mitochondrial respiration and ATP generation in G2 phase (Wang et al., 2014).

Taken together, this study provides clues and methods for further research on the role of CMs in cell cycle progression. Future research is suggested to focus on the molecular regulatory mechanisms underlying the formation and depolymerization of CMs, a more detailed identification of the role of CMs in cell cycle, and their relationship with tumor. The elucidation of these issues will provide us with a more in-depth insight into the role of biomembranes.

## Data Availability Statement

The original contributions presented in the study are included in the article/supplementary material, further inquiries can be directed to the corresponding author/s.

## Author Contributions

HB and CH: conceptualization. DK, RL, JZ, and QZ: methodology. DK: investigation. DK and JL: writing. DK, HB, JL, and RL: funding acquisition. WL and CH: supervision. All authors contributed to the article and approved the submitted version.

## Conflict of Interest

The authors declare that the research was conducted in the absence of any commercial or financial relationships that could be construed as a potential conflict of interest.
